# A multi-country analysis of COVID-19 hospitalizations by vaccination status

**DOI:** 10.1016/j.medj.2023.08.005

**Published:** 2023-11-10

**Authors:** Bronner P. Gonçalves, Waasila Jassat, Joaquín Baruch, Madiha Hashmi, Amanda Rojek, Abhishek Dasgupta, Ignacio Martin-Loeches, Luis Felipe Reyes, Chiara Piubelli, Barbara Wanjiru Citarella, Christiana Kartsonaki, Benjamin Lefèvre, José W. López Revilla, Miles Lunn, Ewen M. Harrison, Moritz U.G. Kraemer, Sally Shrapnel, Peter Horby, Zeno Bisoffi, Piero L. Olliaro, Laura Merson, ISARIC Clinical Characterisation Group, Sheryl Ann Abdukahil, Kamal Abu Jabal, Nashat Abu Salah, Eka Airlangga, Ali Ait Hssain, Chika Akwani, Eman Al Qasim, Angela Alberti, Osama Aldabbourosama, Marta Alessi, Beatrice Alex, Abdulrahman Al-Fares, Jeffrey Aliudin, Mohammed Alkahlout, Lana Almasri, Yousef Al-Saba’a, Rita Alves, Joana Alves Cabrita, Maria Amaral, Phoebe Ampaw, Aditya John Anchan, Andrea Angheben, Yaseen Arabi, Antonio Arcadipane, Patrick Archambault, Lukas Arenz, Rakesh Arora, Elizabeth A. Ashley, Anika Atique, Moad Atlowly, Benjamin Bach, John Kenneth Baillie, J. Kevin Baird, Valeria Balan, Renata Barbalho, Nicholas Yuri Barbosa, Wendy S. Barclay, Michaela Barnikel, Netta Beer, Husna Begum, David Bellemare, Anna Beltrame, Giulia Bertoli, Claudia Bianco, Felwa Bin Humaid, Jonathan Bitton, Catherine Blier, Debby Bogaert, Diogo Borges, Dounia Bouhmani, Thipsavanh Bounphiengsy, Latsaniphone Bountthasavong, Bianca Boxma-de Klerk, Filipa Brás Monteiro, Luca Brazzi, Nina Buchtele, Danilo Buonsenso, Aidan Burrell, Ingrid G. Bustos, Joana Cabrita, Eder Caceres, Rui Caetano Garcês, Josie Campisi, Cecilia Canepa, Janice Caoili, Chiara Simona Cardellino, Filipa Cardoso, Filipe Cardoso, Sofia Cardoso, Gayle Carney, François Martin Carrier, Gail Carson, Mariana Cascão, José Casimiro, Silvia Castañeda, Nidyanara Castanheira, Paolo Cattaneo, Roberta Cavalin, Alexandros Cavayas, Muge Cevik, Bounthavy Chaleunphon, Adrienne Chan, Meera Chand, Anjellica Chen, Matthew Pellan Cheng, Danoy Chommanam, Yock Ping Chow, Nathaniel Christy, Rolando Claure-Del Granado, Sara Clohisey, Cassidy Codan, Marie Connor, Graham S. Cooke, Mary Copland, Amanda Corley, Andrea Cortegiani, Gloria Crowl, Claudina Cruz, Marc Csete, Paula Custodio, Ana da Silva Filipe, Andrew Dagens, Peter Daley, Zaina Dalloul, Heidi Dalton, Jo Dalton, Juliana Damas, Nick Daneman, Emmanuelle A. Dankwa, Jorge Dantas, Frédérick D'Aragon, Cristina De Rose, Thushan de Silva, William Dechert, Emmanuelle Denis, Yael Dishon, k Dhangar, Annemarie B. Docherty, Christl A. Donnelly, Chloe Donohue, Phouvieng Douangdala, James Joshua Douglas, Triona Downer, Mark Downing, Thomas Drake, Murray Dryden, Audrey Dubot-Pérès, Susanne Dudman, Jake Dunning, Mathilde Duplaix, Lucian Durham, Anne Margarita Dyrhol-Riise, Michael Edelstein, Martina Escher, Mariano Esperatti, Catarina Espírito Santo, João Estevão, Amna Faheem, Cameron J. Fairfield, Pedro Faria, Nataly Farshait, Jorge Fernandes, Marília Andreia Fernandes, Joana Ferrão, Mário Ferraz, Bernardo Ferreira, Claudia Figueiredo-Mello, Tom Fletcher, Brigid Flynn, Patricia Fontela, Simon Forsyth, Giuseppe Foti, Robert A. Fowler, Diego Franch-Llasat, Christophe Fraser, John F. Fraser, Ana Freitas Ribeiro, Caren Friedrich, Nora Fuentes, Argin G, Linda Gail Skeie, Carrol Gamble, Rui Garcês, Julia Garcia-Diaz, Esteban Garcia-Gallo, Aisling Gavin, Nuno Germano, Moji Ghadimi, Praveen Kumar Ghisulal, Marco Giani, Jess Gibson, Michelle Girvan, Geraldine Goco, Joan Gómez-Junyent, Margarite Grable, Christopher A. Green, William Greenhalf, Fiona Griffiths, Heidi Gruner, Yusing Gu, Anne-Marie Guerguerian, Daniela Guerreiro, Daniel Haber, Hannah Habraken, Wael Hafez, Matthew Hall, Sophie Halpin, Shaher Hamdan, Raph L. Hamers, Summer Hamza, Hayley Hardwick, Janet Harrison, Alan Hartman, Lars Heggelund, Ross Hendry, Martina Hennessy, Liv Hesstvedt, Dawn Higgins, Rupert Higgins, Samuel Hinton, Antonia Ho, Jan Cato Holter, Juan Pablo Horcajada, Jimmy Ming-Yang Hsu, Abby Hurd, Samreen Ijaz, Carlos Cañada Illana, Hugo Inácio, Mariachiara Ippolito, Tiago Isidoro, Hamza Jaber, Clare Jackson, Denise Jaworsky, Synne Jenum, Philippe Jouvet, Alina Kalicinska, Chris Kandel, Kevin Katz, Aasmine Kaur, Seán Keating, Andrea Kelly, Sadie Kelly, Kalynn Kennon, Sommay Keomany, Imrana Khalid, Michelle E. Kho, Saye Khoo, Peter Kiiza, Beathe Kiland Granerud, Anders Benjamin Kildal, Paul Klenerman, Gry Kloumann Bekken, Stephen R. Knight, Volkan Korten, Caroline Kosgei, Deepali Kumar, Demetrios Kutsogiannis, François Lamontagne, Marina Lanza, Andrew Law, Andy Law, Teresa Lawrence, James Lee, Jennifer Lee, Todd C. Lee, Gary Leeming, Andrew Letizia, Gianluigi Li Bassi, Janet Liang, Wei Shen Lim, Andreas Lind, Samantha Lissauer, Diogo Lopes, Ruth Lyons, Sara Machado, Nimisha Abdul Majeed, Frank Manetta, Ceila Maria Sant`Ana Malaque, Catherine Marquis, Laura Marsh, John Marshall, Alejandro Martín-Quiros, Ana Martins, Caroline Martins Rego, Gennaro Martucci, David Maslove, Christina Matthew, Mayfong Mayxay, Colin McArthur, Anne McCarthy, Rachael McConnochie, Sarah E. McDonald, Allison McGeer, Chris McKay, Kenneth A. McLean, Kusum Menon, Alexander J. Mentzer, António Mesquita, Dan Meyer, Alison M. Meynert, Efstathia Mihelis, Agostinho Monteiro, Giorgia Montrucchio, Sarah Moore, Shona C. Moore, Lina Morales Cely, Lucia Moro, Ben Morton, Caroline Mudara, Mo’nes Muhaisen, Fredrik Müller, Karl Erik Müller, Laveena Munshi, Srinivas Murthy, Dana Mustafa, Dave Nagpal, Mangala Narasimhan, Prashant Nasa, Matthew Nelder, Emily Neumann, Pauline Yeung Ng, Alistair D. Nichol, Lisa Norman, Alessandra Notari, Mahdad Noursadeghi, Dwi Utomo Nusantara, Giovanna Occhipinti, Katie O'Hearn, Larissa Oliveira, David S.Y. Ong, Wilna Oosthuyzen, Peter Openshaw, Massimo Palmarini, Giovanna Panarello, Prasan Kumar Panda, Rachael Parke, Patricia Patricio, Lisa Patterson, Mical Paul, Jorge Paulos, William A. Paxton, Mare Pejkovska, Rui Pereira, Michele Petrovic, Frank Olav Pettersen, Scott Pharand, Ooyanong Phonemixay, Soulichanya Phoutthavong, Maria de Piero, Carlos Pimentel, Catarina Pires, Ayodhia Pitaloka, Riinu Pius, Sergio Poli, Georgios Pollakis, Andra-Maris Post, Diana Póvoas, Jeff Powis, Viladeth Praphasiri, Mark G. Pritchard, Bambang Pujo Semedi, Gregory Purcell, Luisa Quesada, Else Quist-Paulsen, Aldo Rafael, Mutia Rahardjani, José Ramalho, Rajavardhan Rangappa, Indrek Rätsep, Brenda Reeve, Dag Henrik Reikvam, Hongru Ren, Oleksa Rewa, Antonia Ricchiuto, Asgar Rishu, Maria Angelica Rivera Nuñez, Stephanie Roberts, David L. Robertson, Ferran Roche-Campo, Paola Rodari, Bernhard Roessler, Andrea Rossanese, Matteo Rossetti, Clark D. Russell, Aleksander Rygh Holten, Isabela Saba, Musharaf Sadat, Valla Sahraei, Leonardo Salazar, Gabriele Sales, Emely Sanchez, Vanessa Sancho-Shimizu, Gyan Sandhu, Oana Sandulescu, Marlene Santos, Shirley Sarfo-Mensah, Iam Claire E. Sarmiento, Egle Saviciute, Justin Schaffer, Michael Schwameis, Gary Schwartz, Janet T. Scott, James Scott-Brown, Malcolm G. Semple, Tânia Sequeira, Ellen Shadowitz, Anuraj Shankar, Catherine A. Shaw, Victoria Shaw, Dr. Rajesh Mohan Shetty, Bountoy Sibounheuang, Louise Sigfrid, Piret Sillaots, Wai Ching Sin, Dario Sinatti, Mahendra Singh, Vegard Skogen, Sue Smith, Joshua Solomon, Tom Solomon, Rima Song, Elisabetta Spinuzza, Shiranee Sriskandan, Thomas Staudinger, Stephanie-Susanne Stecher, Trude Steinsvik, Birgitte Stiksrud, Adrian Streinu-Cercel, Anca Streinu-Cercel, David Stuart, Decy Subekti, Jacky Y. Suen, Asfia Sultana, Charlotte Summers, Atie Suwarti, Jaques Sztajnbok, Shirin Tabrizi, Sara Taleb, Richard S. Tedder, João Teixeira, Hubert Tessier-Grenier, Shaun Thompson, Emma C. Thomson, Mathew Thorpe, Ryan S. Thwaites, Kristian Tonby, Marta Torre, Rosario Maria Torres Santos-Olmo, Alexis F. Turgeon, Lance C.W. Turtle, Anders Tveita, Pawel Twardowski, Roman Ullrich, Timothy M. Uyeki, Piero Valentini, Luís Val-Flores, Michael Varrone, José Ernesto Vidal, César Vieira, Joy Ann Villanueva, Judit Villar, Andrea Villoldo, Chiara Vitiello, Manivanh Vongsouvath, Marina Wainstein, Steve Webb, Jia Wei, Sanne Wesselius, Murray Wham, Nicole White, Sue Willems, Bailey Williams, Virginie Williams, Evert-Jan Wils, Jessica Wittman, Stephanie Yerkovich, Touxiong Yiaye, Maram Zahran, Maria Zambon

**Affiliations:** 1 ISARIC, Pandemic Sciences Institute, University of Oxford, Oxford, UK; 2 National Institute for Communicable Diseases, Johannesburg, South Africa; 3 Right to Care, Pretoria, South Africa; 4 Critical Care Asia and Ziauddin University, Karachi, Pakistan; 5 Doctoral Training Centre, University of Oxford, Oxford, UK; 6 Department of Biology, University of Oxford, Oxford, UK; 7 Department of Intensive Care Medicine, Multidisciplinary Intensive Care Research Organization (MICRO), St James’s Hospital, Leinster, Dublin, Ireland; 8 Pulmonary Intensive Care Unit, Respiratory Institute, Hospital Clinic of Barcelona, IDIBAPS (Institut d'Investigacions Biomèdiques August Pi i Sunyer), University of Barcelona, CIBERes, Barcelona, Spain; 9 Unisabana Center for Translational Science, School of Medicine, Universidad de La Sabana, Chía, Colombia; 10 Department of Infectious, Tropical Diseases and Microbiology, IRCCS Sacro Cuore Don Calabria Hospital, Negrar di Valpolicella, Verona, Italy; 11 MRC Population Health Research Unit, Clinical Trials Service Unit and Epidemiological Studies Unit, Nuffield Department of Population Health, University of Oxford, Oxford, UK; 12 Université de Lorraine, CHRU-Nancy, Service des Maladies Infectieuses et Tropicales, Nancy, France; 13 Université de Lorraine, APEMAC, Nancy, France; 14 Instituto Nacional del Niño San Borja and Facultad de Ciencias de la Salud, Universidad Científica del Sur, Lima, Peru; 15 Centre for Medical Informatics, The University of Edinburgh, Usher Institute of Population Health Sciences and Informatics, Edinburgh, UK; 16 Pandemic Sciences Institute, University of Oxford, Oxford, UK; 17 Centre for Health Services Research, Faculty of Medicine, The University of Queensland, Herston, Brisbane, Australia; 18 School of Mathematics and Physics, Faculty of Science, The University of Queensland, St Lucia, Brisbane, Australia

**Keywords:** COVID-19, descriptive epidemiology, vaccination, comorbidity, heterogeneity

## Abstract

****Background**:**

Individuals vaccinated against severe acute respiratory syndrome coronavirus 2 (SARS-CoV-2), when infected, can still develop disease that requires hospitalization. It remains unclear whether these patients differ from hospitalized unvaccinated patients with regard to presentation, coexisting comorbidities, and outcomes.

****Methods**:**

Here, we use data from an international consortium to study this question and assess whether differences between these groups are context specific. Data from 83,163 hospitalized COVID-19 patients (34,843 vaccinated, 48,320 unvaccinated) from 38 countries were analyzed.

****Findings**:**

While typical symptoms were more often reported in unvaccinated patients, comorbidities, including some associated with worse prognosis in previous studies, were more common in vaccinated patients. Considerable between-country variation in both in-hospital fatality risk and vaccinated-versus-unvaccinated difference in this outcome was observed.

****Conclusions**:**

These findings will inform allocation of healthcare resources in future surges as well as design of longer-term international studies to characterize changes in clinical profile of hospitalized COVID-19 patients related to vaccination history.

****Funding**:**

This work was made possible by the UK Foreign, Commonwealth and Development Office and Wellcome (215091/Z/18/Z, 222410/Z/21/Z, 225288/Z/22/Z, and 220757/Z/20/Z); the Bill & Melinda Gates Foundation (OPP1209135); and the philanthropic support of the donors to the University of Oxford’s COVID-19 Research Response Fund (0009109). Additional funders are listed in the “acknowledgments” section.

## Highlights

• Vaccinated patients were on average older than unvaccinated individuals
• Hospitalized COVID-19 patients with history of vaccination often had comorbidities
• Fatality risk and vaccinated-versus-unvaccinated difference varied by country

## Context and significance

Although SARS-CoV-2 vaccines are effective and protect against COVID-19, this protection is not perfect, and vaccinated individuals can still develop clinical disease that requires hospitalization. In this international clinical epidemiology study, Gonçalves et al. analyzed data collected in 38 countries to describe characteristics, including coexisting medical conditions, and outcomes of hospitalized COVID-19 patients by vaccination status. Hospitalized patients with history of SARS-CoV-2 vaccination were on average older than hospitalized unvaccinated individuals and more often had multiple comorbidities. There were differences in the risk of death between countries and by vaccination status. These observations will facilitate the design of longer-term studies to assess changes in the profile of COVID-19 patients linked to vaccination and inform better allocation of healthcare resources.

## Graphical abstract

**Figure:**
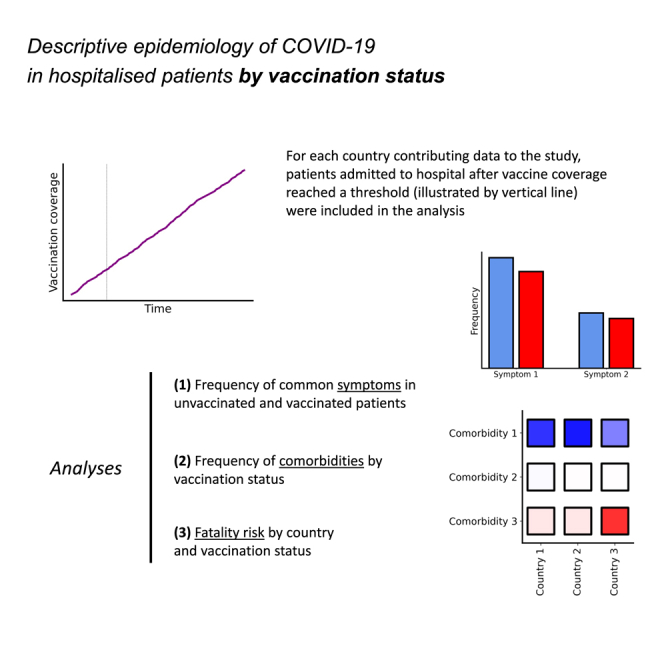

## Teaser

Gonçalves and colleagues analyzed a dataset from a large international clinical consortium to describe presentation, coexisting comorbidities, and outcomes of hospitalized COVID-19 patients by vaccination status. They observed that hospitalized vaccinated patients were on average older, and more often had comorbidities, compared with hospitalized unvaccinated individuals.

## Introduction

The swiftness with which vaccines targeting severe acute respiratory syndrome coronavirus 2 (SARS-CoV-2) were developed and tested^1^^,^^2^^,^^3^ in the first year of the pandemic allowed vaccination to be rolled out as early as December 2020 in some countries. Since then, coverage has increased in all regions, albeit to a variable extent, and millions of lives are estimated to have been saved by immunization programs.^4^ However, despite their effectiveness, current vaccines do not provide sterilizing immunity, and vaccinated individuals can still be infected and develop symptomatic disease. A consequence of this less-than-perfect vaccine-induced immunity, which has been shown to wane over time,^5^^,^^6^ and the increase in coverage is that coronavirus disease 2019 (COVID-19) cases, including those requiring hospital care, often occur in individuals with previous SARS-CoV-2 vaccination. Because disease presentation might differ between vaccinated and unvaccinated patients (for multiple reasons, including non-comparability in terms of prognostic factors linked to the prioritization of high-risk groups for immunization, and vaccine effects on disease progression after infection), the continuing change in the profile of COVID-19 patients with regard to vaccination history means that it is important to systematically describe symptoms, comorbidities, and severity of cases by vaccine status.

Although previous studies^7^^,^^8^^,^^9^^,^^10^^,^^11^ reported on characteristics and outcomes of hospitalized COVID-19 patients with history of SARS-CoV-2 vaccination, these studies often had limited sample sizes or did not allow for between-country comparisons. Here, we analyze data from an international consortium that collected detailed clinical information on hospitalized patients with COVID-19; our objective is to describe presentation, coexisting medical conditions, and outcome of patients admitted to hospital stratified by vaccination status. As this database includes hospital admission records from the beginning of the pandemic until the second half of 2022, to ensure that vaccinated and unvaccinated patients compared here were admitted during the same time period, we restricted analyses to individuals hospitalized after vaccine coverage reached 10% in the corresponding countries.

## Results

### Selection of analytic sample

**Figure 1:**
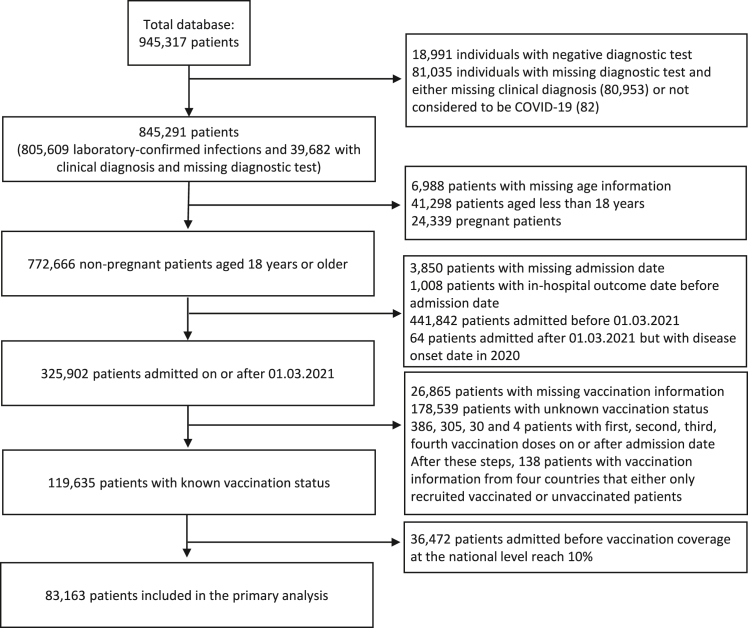
Selection of the analytic sample A database from the Our World in Data initiative with information on country-level vaccination coverage was used in the final step of selection. Pregnant women were excluded from the analysis because previous studies reported that delivery is a common reason for hospital admission in patients with incidental SARS-CoV-2 infections.^12^ See also Figure S1.

The clinical database used in this analysis includes information on 945,317 hospitalized patients from 76 countries; 845,291 either had laboratory-confirmed SARS-CoV-2 infection or were clinically diagnosed with COVID-19 in the absence of a diagnostic test. Data on vaccination were not collected by the consortium before March 2021; for this reason, analyses presented here exclude records of patients admitted to hospital before that month. Furthermore, since population-level vaccine coverage remained low for several months after March 2021 in many of the countries where participating hospitals are located, as mentioned above, only data from patients admitted after 10% of the corresponding country’s population had been vaccinated were included (Figure S1). Sensitivity analyses using a different coverage threshold are described in the Supplementary Appendix and Data S1. Because, in most settings, children and adolescents were offered vaccines after adult age groups, our analysis also excluded patients younger than 18 years of age. In Figure 1, the different steps of the selection of participants for the analysis are presented.

In the next subsections, data from 83,163 non-pregnant adult patients in 38 different countries with known vaccination status are presented. A comparison of these patients with those recruited during the same period but with unknown vaccination history is presented in Data S1. Note that, in some countries, patients requiring clinical management in intensive care units (ICUs) were preferentially enrolled; below, we refer to countries where more than 80% of study participants were admitted to an ICU during hospitalization as the ICU country group (18 countries; Table S1).

### Vaccination data

Most hospital records in the analytic sample were contributed by South Africa and the United Kingdom (47,768 [57.4%] and 29,637 [35.6%]); 2,881 (3.5%) participants were from the ICU country group. Most (71.7%) patients in the analytic sample were admitted in 2021; 28.3% were admitted in 2022. A total of 37,147 (44.7%) patients were hospitalized during the period when the Delta variant was dominant; here, the period during which its frequency at the population level, relative to the other circulating variants, was above 90% (see STAR Methods and Figure S1); 36,976 (44.5%) participants were admitted when the Omicron variant was dominant. Other study participants were admitted either when the Alpha variant caused most infections locally (2,025, 2.4%) or during periods when relative frequencies of all circulating variants were below 90% (7,015, 8.4%).

In the combined dataset, 41.9% (34,843/83,163) of hospitalized patients had been vaccinated. There was between-country variation (Table S2): in the United Kingdom, 64.1% of study participants reported vaccination before hospital admission, while in South Africa and the ICU country group, which includes settings with different population-level vaccination coverages, lower percentages of patients had received SARS-CoV-2 vaccine doses, 27.6% and 34.5% respectively. In some countries, e.g., Norway, there was an increase in the fraction of participants with history of vaccination during the study period (Figure S2), while in others this proportion remained relatively stable or fluctuated non-monotonously.

**Figure 2:**
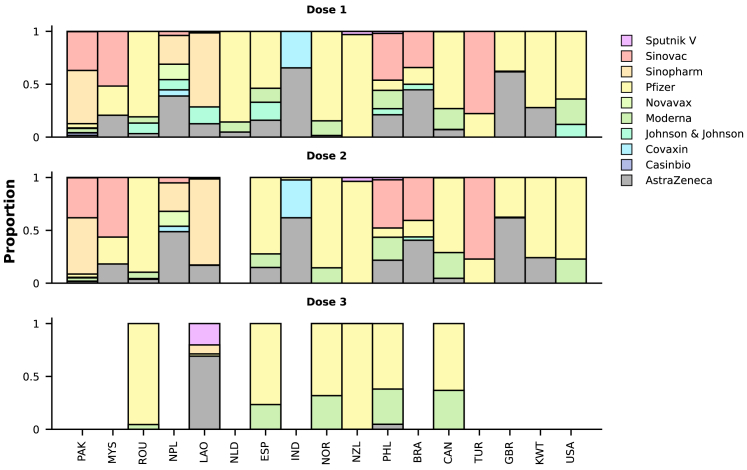
Manufacturers of vaccines administered to study participants The y axes present the distributions of vaccine manufacturers, as proportions, by country (x axes; the same ordering applies to the three panels). For some participants, information on vaccine manufacturer was available for the second or third doses but not for the first dose (N = 25 and 15, respectively) or for the third dose but not the second dose (N = 18). Only countries with at least 20 participants for whom manufacturer information was available are included in this figure; this criterion was also used for each dose-specific panel. Information on vaccine manufacturer for the fourth dose was available for 22 patients and is not presented here. See also Figure S2 and Table S2.

Our comparisons are based on a binary variable corresponding to vaccination history; however, information on the manufacturer of the vaccine used for the first four doses was also available for 78.5% (27,356 out of 34,843), 59.2% (20,618 out of 34,843), 1.6% (562 out of 34,843), and 0.1% (25 out of 34,843) of the vaccinated participants. Note that these numerators include patients who reported “Unknown vaccine type” (10,575, 4,512, 56, and 3, respectively, for the four doses), and the denominators are the same and correspond to the number of individuals with previous vaccination, rather than to those reporting different numbers of previous vaccine doses, as this information was not captured in the case report form. In the combined data, for first doses, the most frequently used vaccines were those produced by AstraZeneca (55.3%, 9,275 out of 16,781) and Pfizer (37.5%, 6,294 out of 16,781); distributions of vaccine manufacturers by country and dose are shown in Figure 2. Of patients with data on the first two doses, most received the same vaccine type in the first and second doses (16,048 out of 16,097).

Information on dates when patients received vaccine doses was available for 16,036, 13,312, and 500 participants for the first three doses. The median time interval between the first dose and hospital admission was 236 days (interquartile range [IQR] 157–306; N = 16,036), and between the most recent dose and admission was 172 days (IQR 104–237; N = 13,985). Of participants with data on vaccination dates, 2.6% (424 out of 16,036) were admitted in the 2 weeks following the first dose; this percentage was higher (3.4%) for patients admitted in 2021 compared with those admitted in 2022 (0.3%). For patients recruited in the ICU country group, median time intervals from the first and the most recent dose to hospital admission were, respectively, 165 (IQR 77–256) and 110 days (IQR 42–187). When calculating time from the most recent dose, patients for whom vaccination date was missing for a later vaccine dose with manufacturer information were not included.

### Age and symptoms by vaccination status

In many settings, individuals at higher risk of severe disease were prioritized for vaccination. This initial susceptibility-based allocation of SARS-CoV-2 vaccines, aimed at maximizing public health impact, implies that vaccinated and unvaccinated patients are expected to differ in many clinically relevant characteristics. Consistent with this, although similar percentages of vaccinated and unvaccinated patients were male (50.0% and 48.0%, respectively), the median (IQR) age of those vaccinated was 65 (50–78) years, higher than the median age of unvaccinated patients (50 [35–65] years). In Table S3, median, and corresponding IQR, ages of vaccinated and unvaccinated participants are presented by country; in most countries, vaccinated patients were older than unvaccinated patients.

**Figure 3:**
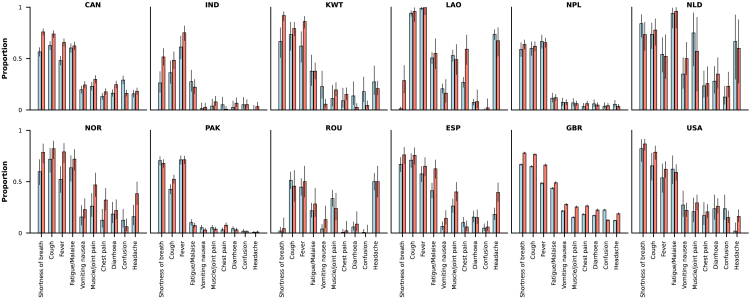
Country-specific frequencies of the 10 most common symptoms Red bars correspond to data from unvaccinated patients and light blue bars to data from vaccinated patients. The ordering of symptoms (x axes) is the same in all panels; y axes present frequencies as proportions. The 95% confidence intervals are also shown. Only countries with at least 100 patients with data on one or more symptoms are included; the ISO3 code of each country included is presented as the corresponding panel title: CAN, Canada; IND, India; KWT, Kuwait; LAO, Laos; NPL, Nepal; NLD, the Netherlands; NOR, Norway; PAK, Pakistan; ROU, Romania; ESP, Spain; GBR, United Kingdom; USA, United States. See Table S4.

Data on symptoms were available for 30,341 out of 35,395 individuals; the denominator here does not include South African patients, as this information was not available for participants from that country. The analysis included variables on 24 different symptoms, and the median (IQR) number of symptoms with non-missing information per patient was 23 (19–24). Table S4 shows the frequencies of the different symptoms by vaccination status; some of the most common symptoms, e.g., fever, shortness of breath, and cough, were less frequent in the vaccinated group. Among patients with non-missing information on the five most common symptoms in the dataset (shortness of breath, cough, fever, fatigue/malaise, vomiting/nausea), 90.9% (12,041 out of 13,251) of those with history of vaccination had at least one of these symptoms; the percentage of unvaccinated patients with one or more of these typical symptoms was 95.6% (9,630 out of 10,070). Country-specific frequencies of symptoms by vaccination status are shown in Figure 3; data for most countries included in the figure suggest slightly higher frequencies in the unvaccinated compared with the vaccinated group. An exception to this pattern is the frequency of confusion or altered consciousness, which was more often seen in vaccinated patients. Table S4 presents the comparison by SARS-CoV-2 variant period.

### Comorbidities in vaccinated and unvaccinated patients

**Figure 4:**
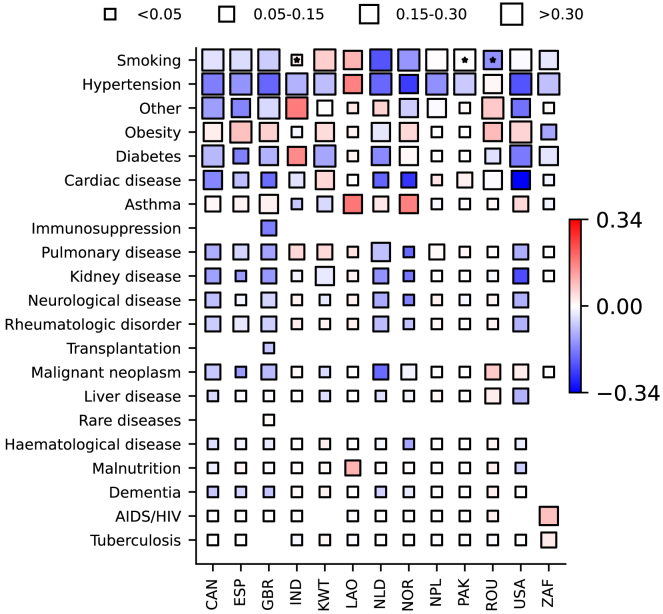
Differences in frequencies of comorbidities between vaccinated and unvaccinated groups Coordinates in the y axis correspond to comorbidities, ordered based on mean frequencies in all countries, and in the x axis, countries, represented by ISO3 codes, are ordered alphabetically. The four different sizes of the squares in the figure relate to the corresponding frequencies in the unvaccinated group (see top of the graph). Colors represent country- and comorbidity-specific numerical differences in frequencies between unvaccinated and vaccinated patients; red tones indicate that a comorbidity was more frequent in unvaccinated patients. Only countries with 100 or more patients with data on at least one comorbidity are presented. Stars indicate when the number of vaccinated or unvaccinated individuals was below 20. A different version of this figure is shown in the Supplementary Appendix (Figure S3) that accounts for frequencies of comorbidities in the unvaccinated group not only in the size of the squares but also in the color; i.e., the other version of this figure presents differences relative to the frequencies in the unvaccinated group rather than absolute differences.

A total of 21 variables on specific comorbidities were analyzed; information on at least one of these variables was available for most participants (76,892 out of 83,163; 92.3% and 92.6% of vaccinated and unvaccinated individuals). The median number of comorbidities in patients with data on at least one comorbidity was two (IQR, 1–3) for the vaccinated group, and one (IQR, 0–2) for the unvaccinated group. Frequencies of the different medical conditions are presented in Table 1; several, e.g., chronic cardiac, pulmonary and kidney conditions, were more often reported in vaccinated compared with unvaccinated patients (respectively, 22.1% versus 7.1%, 13.7% versus 4.3%, 14.1% versus 3.7%). As both vaccination coverage and prevalence of comorbidities vary by country, we also analyzed country-specific data on coexisting conditions. In Figure 4, differences in frequencies of comorbidities between vaccinated and unvaccinated individuals are presented. Some conditions were more common in patients with a history of SARS-CoV-2 vaccination: for example, for several countries, hypertension and chronic cardiac disease were more frequent in the vaccinated group.

**Table 1:** Frequencies of comorbidities in vaccinated and unvaccinated patients in the combined (all-country) dataset

Comorbidities	Vaccinated			Unvaccinated		
	%	Total (non-missing)	Missing data	%	Total (non-missing)	Missing data
AIDS/HIV	3.1	27,675	7,168	11.6	31,369	16,951
Asthma	11.9	28,421	6,422	7.4	32,532	15,788
Cardiac disease	22.1	28,480	6,363	7.1	32,412	15,908
Hematological disease	4.9	17,836	17,007	2.3	9,542	38,778
Kidney disease	14.1	28,280	6,563	3.7	32,067	16,253
Neurological disease	11.6	18,006	16,837	5.9	9,670	38,650
Pulmonary disease	13.7	28,386	6,457	4.3	32,102	16,218
Dementia	10.3	17,776	17,067	3.1	9,585	38,735
Diabetes	27.7	28,640	6,203	18.9	33,870	14,450
Hypertension	47.8	29,426	5,417	33.6	35,145	13,175
Immunosuppression	24.8	2,741	32,102	7.7	901	47,419
Liver disease	3.8	18,917	15,926	3.1	10,085	38,235
Malignant neoplasm	8.9	28,213	6,630	2.1	32,136	16,184
Malnutrition	1.7	16,732	18,111	1.4	9,285	39,035
Obesity	17.2	19,120	15,723	15.7	21,530	26,790
Other	28.7	30,251	4,592	10.8	43,344	4,976
Rare diseases	1.7	2,845	31,998	2.4	915	47,405
Rheumatologic disorder	12.0	17,801	17,042	5.6	9,524	38,796
Smoking	46.7	13,261	21,582	26.3	17,229	31,091
Transplantation	12.2	2,853	31,990	4.3	915	47,405
Tuberculosis	2.2	12,676	22,167	4.8	25,165	23,155

See also Table S5.

The presence of multiple comorbidities was not infrequent in the analytic sample. Of the 58,330 participants with non-missing data on at least 10 comorbidities, 27.9% had three or more comorbidities. Of the vaccinated individuals, 42.0% (11,584 out of 27,571) had a medical history of three or more comorbidities, compared with 15.2% (4,692 out of 30,759) of the unvaccinated patients. A similar pattern was observed in each age group: for patients aged between 18 and 60 years, the frequency of three or more comorbidities was 20.4% (2,082 out of 10,198) and 10.4% (1,984 out of 19,105) for those with history of vaccination and those without previous vaccination, respectively; for patients older than 60 years, these percentages were respectively 54.7% (9,502 out of 17,373) and 23.2% (2,708 out of 11,654). For the ICU country group, the frequencies of multiple comorbidities in vaccinated and unvaccinated patients were 13.5% (132 out of 980) and 14.8% (274 out of 1,846), respectively. Table 2 presents country-specific data, and frequencies of multiple comorbidities for different variant periods are shown in Table S5.

**Table 2:** Percentages of patients with three or more comorbidities by vaccination status and country

Country	Vaccinated		Unvaccinated	
	% with three or more comorbidities	Total	% with three or more comorbidities	Total
Canada	63.0	562	40.0	697
India	10.0	80	13.7	153
Kuwait	26.7	45	28.8	163
Lao PDR	0.6	344	14.3	49
Nepal	5.5	219	4.6	461
The Netherlands	64.7	68	30.3	66
Norway	58.6	70	23.3	86
Pakistan	1.0	483	1.3	603
Romania	12.5	152	15.2	46
South Africa	7.6	9,502	7.4	21,076
Spain	50.8	187	28.0	125
United Kingdom	65.3	15,450	37.1	6,642
United States	78.6	56	50.9	175

Only participants with non-missing information on 10 or more comorbidity-related variables were included; information is presented for countries with at least 100 patients meeting this criterion. See also Tables S7–S10.

### In-hospital outcomes

**Figure 5:**
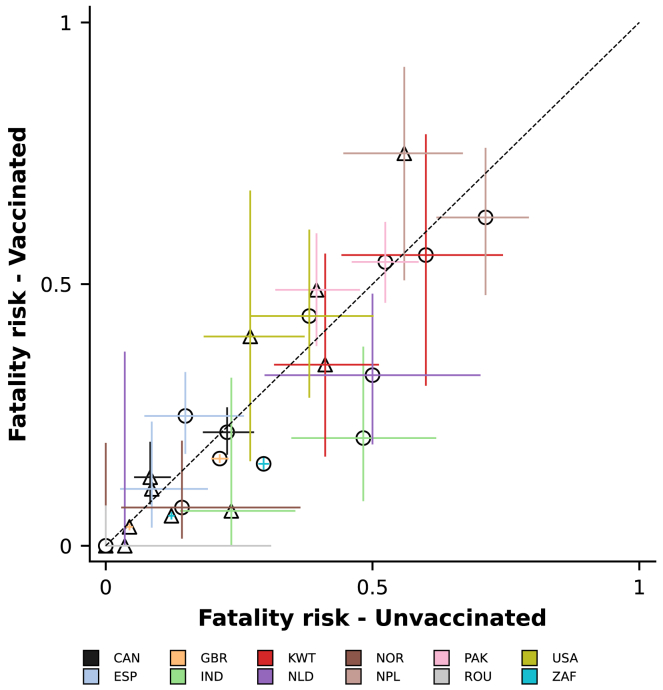
Fatality risks in vaccinated (y axis) and unvaccinated (x axis) patients by country, represented by different colors, and age Patients were grouped in two broad age categories for this figure: those aged between 18 and 60 years, represented by triangles, and patients older than 60 years, represented by circles. The 95% confidence intervals for both the vaccinated (vertical lines) and unvaccinated (horizontal lines) groups are shown. Figure S4 presents a version of this figure where the two axes range from 0 to 0.30, allowing better visualization of data from countries with lower fatality risks, including South Africa and the United Kingdom. See also Tables S7 and S8.

We compared the risk of death during the first 28 days since admission or disease onset, whichever happened later, for vaccinated and unvaccinated patients (see STAR Methods for more details on the definition of the outcome). All data combined, there were 4,053 (12.4%) deaths in the vaccinated group, and, in the unvaccinated group, 7,832 out of 46,170 (17.0%) patients died. The fatality risk was higher in participants aged 60 years or older (17.1% [3,333 out of 19,432] and 28.8% [4,516 out of 15,698] in the vaccinated and unvaccinated groups, respectively) compared with younger patients, aged from 18 to 60 years (5.5% [720 out of 13,167] and 10.9% [3,316 out of 30,472] in the vaccinated and unvaccinated groups). Patients recruited in the ICU country group also had higher death risk, 38.9% (81 out of 208) and 33.1% (230 out of 694), respectively, for vaccinated and unvaccinated patients aged from 18 to 60 years, and 47.9% (185 out of 386) and 51.2% (353 out of 689) for vaccinated and unvaccinated patients aged 60 years or older. Country-specific data on the risk of death are presented in Table S5, and Figure 5 shows fatality risk by country, age, and vaccination group: while, in some countries, e.g., the United Kingdom and South Africa, unvaccinated patients had higher risk of death, in others, with more limited study sample size, a higher percentage of vaccinated patients died compared with unvaccinated patients. A multivariable analysis, adjusting for sex, age, and number of comorbidities, was also performed and the odds ratio estimated for the association between vaccination and death was 0.53 (95% confidence interval 0.50–0.56; Table S6). A *post hoc* regression analysis was also performed excluding data from the ICU country group; similar results were obtained (odds ratio 0.50, 0.47–0.53).

In addition to death, we also analyzed a composite outcome combining death and invasive mechanical ventilation. In analyses restricted to countries where less than 80% of the analytic sample required ICU admission, the composite outcome was more frequent in unvaccinated patients; in the ICU country group, the frequency of the composite outcome was high (>85%) regardless of vaccination status or age. Results of this analysis are detailed in the Supplementary Appendix and Data S1.

## Discussion

Among the many important contributions of epidemiology to the pandemic response are studies on the effectiveness of SARS-CoV-2 vaccines.^13^^,^^14^^,^^15^^,^^16^ These have been instrumental to generate evidence on the effect of vaccines against different variants and in settings different from those where clinical trials were performed. These studies were often designed as test-negative studies or population-based studies that use electronic medical records. Our study has a different design; here, only patients who developed disease severe enough to require hospital admission were included, and non-COVID-19 patients were not recruited as part of this consortium. Hence, we did not aim to quantify vaccine effectiveness against infection or hospitalization; we rather aimed to describe the clinical profile of hospitalized COVID-19 patients by vaccination status. In the “results” section, we reported clinical data from 83,163 patients from 38 countries. Our analyses show that hospitalized vaccinated individuals were on average older than hospitalized unvaccinated individuals and more often had multiple comorbidities. Despite the variable fatality risk observed in different countries, descriptive analyses indicate that history of vaccination is associated with lower death risk in some settings despite initial prioritization of high-risk group for vaccination. Although these findings suggest differences between these groups, more consistently with regard to coexisting medical conditions, as vaccine coverage, including of booster doses, increases, a finer comparison based on time since most recent dose might be required to aid clinical practice and allocation of healthcare resources. The data presented here, in particular on the between-country heterogeneity in patient characteristics, will inform design of future international studies on this question.

The observation that, in the population of hospitalized COVID-19 patients, vaccinated individuals were on average older than unvaccinated individuals was expected given that, in many settings, age was used in prioritizing the initial allocation of vaccine doses. However, in some countries, this pattern persisted during the entire study period: for example, in the United Kingdom, participants aged 60 years or older represented 70.3% of the vaccinated patients and 29.2% of the unvaccinated patients in 2021 and 75.5% and 50.4%, respectively, in 2022; in South Africa, vaccinated patients were also more often older than 60 years compared with unvaccinated patients in both calendar years (see Table S3 for other similar country-specific comparisons). One possible explanation is that collider bias,^17^ due to selection of patients with severe disease, i.e., patients requiring hospital admission, might have contributed to this pattern. Collider bias^18^ can occur when study selection is affected by the two factors being studied or by their causes. Because both previous SARS-CoV-2 vaccination and age affect disease severity, and consequently risk of hospitalization, an association between these two factors is expected in the hospitalized population. Other explanations could relate to non-optimal immunological response to vaccination^19^^,^^20^^,^^21^ or higher immunization uptake in older age groups.

One of the effects of SARS-CoV-2 vaccines is on clinical progression after viral infection,^22^ and it seems reasonable to assume that this might also influence frequencies of symptoms, even among those individuals with severe disease. In our analysis, we observed that typical symptoms were more often present in unvaccinated than vaccinated individuals, although the difference was not substantial. The inclusion of patients with incidental infections might have contributed to this observation. In hospital-based studies, incidental infections complicate quantitative descriptions of clinical disease as, for patients with these infections, COVID-19 is not the primary cause of hospitalization, and COVID-19-related symptoms are thus not necessarily present, or rather, by definition, are less likely to be present. As previous studies suggest that delivery is a common cause of hospitalization in patients with incidental SARS-CoV-2 infections, pregnant women were excluded from this analysis (Figure 1). Relative frequencies of different variants should also be considered when interpreting these results, as a recent study provided evidence that infections caused by different variants could be associated with different symptoms.^23^ As mentioned in the “results” section, unlike most other common symptoms, confusion or altered consciousness was more often observed in vaccinated individuals; it is possible that this relates to differences in age distribution between the vaccinated and unvaccinated groups.^24^

The vaccinated and unvaccinated groups also differed in terms of comorbidities. In several countries, some of the most frequent comorbidities, e.g., hypertension and cardiac disease, were more prevalent in vaccinated patients. This pattern was also observed in analyses performed by SARS-CoV-2 variant periods. The two mechanisms described above, prioritization of high-risk groups for vaccination and potential collider bias, are likely to have contributed to this observation, which is consistent with a few previous hospital-based studies.^25^ For example, in a study from the United States,^26^ vaccinated patients admitted to hospital, in addition to being older than unvaccinated patients, more often had comorbidities; in an Israeli study,^27^ comorbidities were also more frequent in vaccinated versus unvaccinated patients. Epidemiological studies, including some performed before vaccine introduction,^28^^,^^29^ estimated that many of these conditions are associated with COVID-19-related death; this imbalance in coexisting comorbidities between vaccinated and unvaccinated patients might thus influence in-hospital prognosis for these patients. A limitation of this analysis is that only information on the presence or absence of specific comorbidities was collected, but not on their relative severities, which would have helped to understand their clinical significance for hospital admission. Note that, in South Africa, vaccinated and unvaccinated patients had similar frequencies of multiple comorbidities; it is unclear whether this relates to local epidemiology and transmission patterns, to a higher proportion of incidental infections, or to data missingness (for this country, only 12 out of 21 comorbidity-related variables were available for analysis).

Previous studies reported conflicting results regarding the risk of death in vaccinated and unvaccinated patients hospitalized with COVID-19. In the study conducted by Mielke and colleagues, fewer vaccinated individuals died (10.3%) compared with unvaccinated patients (12.8%).^11^ However, in other studies,^26^^,^^27^ similar fatality risks were observed in these groups. In our analysis, the odds ratio for the association between previous vaccination and death in the combined dataset, dominated by data from South Africa and the United Kingdom, suggests vaccinated patients had a lower risk of death. There was, however, considerable between-country variation both in the overall risk of death and in the differences between vaccinated and unvaccinated individuals (Figure 5). While for patients recruited in the United Kingdom, South Africa, and India, the risk of death was lower in the vaccinated group, for countries that might have primarily recruited more severely ill patients, e.g., Pakistan and the United States, the opposite pattern was observed. More generally, differences in fatality risk between vaccinated and unvaccinated hospitalized patients should not be interpreted as measures of vaccine effectiveness: both confounding due to vaccine prioritization and collider bias due to study sample selection can affect associations between vaccination and disease severity in these analyses, but, foremost, these estimates do not take into account the components of the vaccine-induced protection against infection and against progression to disease that requires hospital care conditional on infection.

Given the increasing coverage of vaccination, it is possible that the next surge of SARS-CoV-2 infections will affect primarily vaccinated individuals. Our analysis, which used data from one of the largest cohorts of hospitalized COVID-19 patients, suggests that, when these individuals are infected, develop disease, and are admitted to hospital, they might be more likely to have comorbidities and be older than patients with no previous vaccination. However, it does not necessarily follow that the same pattern will be observed in comparisons of vaccinated individuals with versus without booster doses, and international studies with design similar to ours and that are able to more comprehensively capture information on vaccination dates will be informative in the next waves of this pandemic.

### Limitations of the study

The main strength of our study is the joint description of data from more than one country, which allowed assessment of the consistency of findings in countries with different SARS-CoV-2 epidemiological trajectories and vaccine coverage. On the other hand, the high proportion of hospitalizations with missing data on previous vaccination is an important weakness of our analysis. In Data S1, we present comparisons between participants for whom information on vaccination status was available versus those with missing vaccine data; we did not observe clear differences between these groups in aggregated analyses. Information on dates when vaccine doses were administered was also incomplete, only available for 46.0% of first doses in patients reporting previous vaccination. Another aspect of our analysis that should be addressed in future studies on similar questions relates to the identification of incidental SARS-CoV-2 infections as these might affect comparisons, especially if their frequency is influenced by vaccination. Finally, the inclusion of patients with more severe presentation in some countries is both a weakness and a strength; while it increases heterogeneity in the study population, complicating interpretation of aggregated summaries, we were able to describe data from this group of countries and compare with the combined dataset.

## Consortia

The members of the ISARIC Clinical Characterisation Group include Sheryl Ann Abdukahil, Kamal Abu Jabal, Nashat Abu Salah, Eka Airlangga, Ali Ait Hssain, Chika Akwani, Eman Al Qasim, Angela Alberti, Osama Aldabbourosama, Marta Alessi, Beatrice Alex, Abdulrahman Al-Fares, Jeffrey Aliudin, Mohammed Alkahlout, Lana Almasri, Yousef Al-Saba’a, Rita Alves, Joana Alves Cabrita, Maria Amaral, Phoebe Ampaw, Aditya John Anchan, Andrea Angheben, Yaseen Arabi, Antonio Arcadipane, Patrick Archambault, Lukas Arenz, Rakesh Arora, Elizabeth A. Ashley, Anika Atique, Moad Atlowly, Benjamin Bach, John Kenneth Baillie, J. Kevin Baird, Valeria Balan, Renata Barbalho, Nicholas Yuri Barbosa, Wendy S. Barclay, Michaela Barnikel, Netta Beer, Husna Begum, David Bellemare, Anna Beltrame, Giulia Bertoli, Claudia Bianco, Felwa Bin Humaid, Jonathan Bitton, Catherine Blier, Debby Bogaert, Diogo Borges, Dounia Bouhmani, Thipsavanh Bounphiengsy, Latsaniphone Bountthasavong, Bianca Boxma-de Klerk, Filipa Brás Monteiro, Luca Brazzi, Nina Buchtele, Danilo Buonsenso, Aidan Burrell, Ingrid G. Bustos, Joana Cabrita, Eder Caceres, Rui Caetano Garcês, Josie Campisi, Cecilia Canepa, Janice Caoili, Chiara Simona Cardellino, Filipa Cardoso, Filipe Cardoso, Sofia Cardoso, Gayle Carney, François Martin Carrier, Gail Carson, Mariana Cascão, José Casimiro, Silvia Castañeda, Nidyanara Castanheira, Paolo Cattaneo, Roberta Cavalin, Alexandros Cavayas, Muge Cevik, Bounthavy Chaleunphon, Adrienne Chan, Meera Chand, Anjellica Chen, Matthew Pellan Cheng, Danoy Chommanam, Yock Ping Chow, Nathaniel Christy, Rolando Claure-Del Granado, Sara Clohisey, Cassidy Codan, Marie Connor, Graham S. Cooke, Mary Copland, Amanda Corley, Andrea Cortegiani, Gloria Crowl, Claudina Cruz, Marc Csete, Paula Custodio, Ana da Silva Filipe, Andrew Dagens, Peter Daley, Zaina Dalloul, Heidi Dalton, Jo Dalton, Juliana Damas, Nick Daneman, Emmanuelle A. Dankwa, Jorge Dantas, Frédérick D'Aragon, Cristina De Rose, Thushan de Silva, William Dechert, Emmanuelle Denis, Yael Dishon, k Dhangar, Annemarie B. Docherty, Christl A. Donnelly, Chloe Donohue, Phouvieng Douangdala, James Joshua Douglas, Triona Downer, Mark Downing, Thomas Drake, Murray Dryden, Audrey Dubot-Pérès, Susanne Dudman, Jake Dunning, Mathilde Duplaix, Lucian Durham III, Anne Margarita Dyrhol-Riise, Michael Edelstein, Martina Escher, Mariano Esperatti, Catarina Espírito Santo, João Estevão, Amna Faheem, Cameron J. Fairfield, Pedro Faria, Nataly Farshait, Jorge Fernandes, Marília Andreia Fernandes, Joana Ferrão, Mário Ferraz, Bernardo Ferreira, Claudia Figueiredo-Mello, Tom Fletcher, Brigid Flynn, Patricia Fontela, Simon Forsyth, Giuseppe Foti, Robert A. Fowler, Diego Franch-Llasat, Christophe Fraser, John F. Fraser, Ana Freitas Ribeiro, Caren Friedrich, Nora Fuentes, Argin G, Linda Gail Skeie, Carrol Gamble, Rui Garcês, Julia Garcia-Diaz, Esteban Garcia-Gallo, Aisling Gavin, Nuno Germano, Moji Ghadimi, Praveen Kumar Ghisulal, Marco Giani, Jess Gibson, Michelle Girvan, Geraldine Goco, Joan Gómez-Junyent, Margarite Grable, Christopher A. Green, William Greenhalf, Fiona Griffiths, Heidi Gruner, Yusing Gu, Anne-Marie Guerguerian, Daniela Guerreiro, Daniel Haber, Hannah Habraken, Wael Hafez, Matthew Hall, Sophie Halpin, Shaher Hamdan, Raph L. Hamers, Summer Hamza, Hayley Hardwick, Janet Harrison, Alan Hartman, Lars Heggelund, Ross Hendry, Martina Hennessy, Liv Hesstvedt, Dawn Higgins, Rupert Higgins, Samuel Hinton, Antonia Ho, Jan Cato Holter, Juan Pablo Horcajada, Jimmy Ming-Yang Hsu, Abby Hurd, Samreen Ijaz, Carlos Cañada Illana, Hugo Inácio, Mariachiara Ippolito, Tiago Isidoro, Hamza Jaber, Clare Jackson, Denise Jaworsky, Synne Jenum, Philippe Jouvet, Alina Kalicinska, Chris Kandel, Kevin Katz, Aasmine Kaur, Seán Keating, Andrea Kelly, Sadie Kelly, Kalynn Kennon, Sommay Keomany, Imrana Khalid, Michelle E. Kho, Saye Khoo, Peter Kiiza, Beathe Kiland Granerud, Anders Benjamin Kildal, Paul Klenerman, Gry Kloumann Bekken, Stephen R. Knight, Volkan Korten, Caroline Kosgei, Deepali Kumar, Demetrios Kutsogiannis, François Lamontagne, Marina Lanza, Andrew Law, Andy Law, Teresa Lawrence, James Lee, Jennifer Lee, Todd C. Lee, Gary Leeming, Andrew Letizia, Gianluigi Li Bassi, Janet Liang, Wei Shen Lim, Andreas Lind, Samantha Lissauer, Diogo Lopes, Ruth Lyons, Sara Machado, Nimisha Abdul Majeed, Frank Manetta, Ceila Maria Sant`Ana Malaque, Catherine Marquis, Laura Marsh, John Marshall, Alejandro Martín-Quiros, Ana Martins, Caroline Martins Rego, Gennaro Martucci, David Maslove, Christina Matthew, Mayfong Mayxay, Colin McArthur, Anne McCarthy, Rachael McConnochie, Sarah E. McDonald, Allison McGeer, Chris McKay, Kenneth A. McLean, Kusum Menon, Alexander J. Mentzer, António Mesquita, Dan Meyer, Alison M. Meynert, Efstathia Mihelis, Agostinho Monteiro, Giorgia Montrucchio, Sarah Moore, Shona C. Moore, Lina Morales Cely, Lucia Moro, Ben Morton, Caroline Mudara, Mo’nes Muhaisen, Fredrik Müller, Karl Erik Müller, Laveena Munshi, Srinivas Murthy, Dana Mustafa, Dave Nagpal, Mangala Narasimhan, Prashant Nasa, Matthew Nelder, Emily Neumann, Pauline Yeung Ng, Alistair D. Nichol, Lisa Norman, Alessandra Notari, Mahdad Noursadeghi, Dwi Utomo Nusantara, Giovanna Occhipinti, Katie O'Hearn, Larissa Oliveira, David S.Y. Ong, Wilna Oosthuyzen, Peter Openshaw, Massimo Palmarini, Giovanna Panarello, Prasan Kumar Panda, Rachael Parke, Patricia Patricio, Lisa Patterson, Mical Paul, Jorge Paulos, William A. Paxton, Mare Pejkovska, Rui Pereira, Michele Petrovic, Frank Olav Pettersen, Scott Pharand, Ooyanong Phonemixay, Soulichanya Phoutthavong, Maria de Piero, Carlos Pimentel, Catarina Pires, Ayodhia Pitaloka, Riinu Pius, Sergio Poli, Georgios Pollakis, Andra-Maris Post, Diana Póvoas, Jeff Powis, Viladeth Praphasiri, Mark G. Pritchard, Bambang Pujo Semedi, Gregory Purcell, Luisa Quesada, Else Quist-Paulsen, Aldo Rafael, Mutia Rahardjani, José Ramalho, Rajavardhan Rangappa, Indrek Rätsep, Brenda Reeve, Dag Henrik Reikvam, Hongru Ren, Oleksa Rewa, Antonia Ricchiuto, Asgar Rishu, Maria Angelica Rivera Nuñez, Stephanie Roberts, David L. Robertson, Ferran Roche-Campo, Paola Rodari, Bernhard Roessler, Andrea Rossanese, Matteo Rossetti, Clark D. Russell, Aleksander Rygh Holten, Isabela Saba, Musharaf Sadat, Valla Sahraei, Leonardo Salazar, Gabriele Sales, Emely Sanchez, Vanessa Sancho-Shimizu, Gyan Sandhu, Oana Sandulescu, Marlene Santos, Shirley Sarfo-Mensah, Iam Claire E. Sarmiento, Egle Saviciute, Justin Schaffer, Michael Schwameis, Gary Schwartz, Janet T. Scott, James Scott-Brown, Malcolm G. Semple, Tânia Sequeira, Ellen Shadowitz, Anuraj Shankar, Catherine A. Shaw, Victoria Shaw, Dr. Rajesh Mohan Shetty, Bountoy Sibounheuang, Louise Sigfrid, Piret Sillaots, Wai Ching Sin, Dario Sinatti, Mahendra Singh, Vegard Skogen, Sue Smith, Joshua Solomon, Tom Solomon, Rima Song, Elisabetta Spinuzza, Shiranee Sriskandan, Thomas Staudinger, Stephanie-Susanne Stecher, Trude Steinsvik, Birgitte Stiksrud, Adrian Streinu-Cercel, Anca Streinu-Cercel, David Stuart, Decy Subekti, Jacky Y. Suen, Asfia Sultana, Charlotte Summers, Atie Suwarti, Jaques Sztajnbok, Shirin Tabrizi, Sara Taleb, Richard S. Tedder, João Teixeira, Hubert Tessier-Grenier, Shaun Thompson, Emma C. Thomson, Mathew Thorpe, Ryan S. Thwaites, Kristian Tonby, Marta Torre, Rosario Maria Torres Santos-Olmo, Alexis F. Turgeon, Lance C.W. Turtle, Anders Tveita, Pawel Twardowski, Roman Ullrich, Timothy M. Uyeki, Piero Valentini, Luís Val-Flores, Michael Varrone, José Ernesto Vidal, César Vieira, Joy Ann Villanueva, Judit Villar, Andrea Villoldo, Chiara Vitiello, Manivanh Vongsouvath, Marina Wainstein, Steve Webb, Jia Wei, Sanne Wesselius, Murray Wham, Nicole White, Sue Willems, Bailey Williams, Virginie Williams, Evert-Jan Wils, Jessica Wittman, Stephanie Yerkovich, Touxiong Yiaye, Maram Zahran, and Maria Zambon.

## STAR★Methods

### Key resources table

**Table:** 

REAGENT or RESOURCE	SOURCE		IDENTIFIER
**Deposited data**			

Code – descriptive analyses in text		https://doi.org/10.5281/zenodo.8252909; https://github.com/BronnerG/Hosp_Vacc_analysis	

**Software and algorithms**			

Stata version 17	Stata Corp, Texas, TX, USA	https://www.stata.com/	
Python version 3.7	Python Software Foundation	https://www.python.org/	

### Resource availability

#### Lead contact

Further information should be directed to and will be fulfilled by the lead contact, Bronner P. Gonçalves (bronnergoncalves@gmail.com).

#### Materials availability

The study did not generate any new reagents or materials.

### Experimental model and study participant details

Data on clinical presentation and outcomes of hospitalised COVID-19 patients were analyzed in this study. Information on the patients’ age and sex is reported in the results section. These data are part of the ISARIC (International Severe Acute Respiratory and Emerging Infections Consortium) COVID-19 database, and were collected using standardised case report forms, and are stored at a central repository at the University of Oxford. Additional information on ISARIC can be found here https://isaric.org/about-us/membership/, and on data collection and curation can be found in other publications.^30^^,^^31^

### Method details

#### Population-level data on vaccination and SARS-CoV-2 variants

We used data available in the website https://ourworldindata.org/on country-level vaccination coverage^32^ to restrict analyses to patients hospitalised after 10% of a country population had been vaccinated; data were downloaded on April 7, 2023. The objective was to report on a more clinically relevant comparison, i*.*e., of vaccinated and unvaccinated patients admitted to hospital during the same period, rather than on a comparison that would reflect mostly vaccine prioritisation of high-risk groups and temporal changes in clinical prognosis independent of vaccination.

We also stratified key analyses by periods defined based on the relative frequencies of SARS-CoV-2 variants. Data from the Global Initiative on Sharing All Influenza Data (GISAID) on each of the main SARS-CoV-2 variants were aggregated by sample collection date (epidemiologic week) using a bespoke pipeline available from here https://github.com/globaldothealth/covid19-variants-summary. GISAID data were downloaded on October 10, 2022. Country- and variant-specific periods were defined based on the dates when the relative frequency of the temporarily dominant variant was first above and subsequently below 90%; only epidemiological weeks with 10 or more samples in GISAID were analyzed, and after the start of each period, drops to frequencies between 80 and 90% were not considered in defining the end of the period. Note that information on country-level frequencies of variants was not available for the entire study period for some of the countries contributing data to the analytic sample.

### Quantification and statistical analysis

Our aim was to compare clinical presentation, existing comorbidities, and disease progression after admission of vaccinated and unvaccinated patients hospitalised with COVID-19. Count and discretised continuous variables such as age were summarised with medians and interquartile ranges; categorical variables were presented using frequencies, proportions or percentages, of observations in each stratum.

In addition to frequencies of symptoms and comorbidities, we also analyzed risk of in-hospital death. For this, only data from patients still in hospital 28 days after admission or disease onset, regardless of their subsequent outcome, those discharged, and patients who died in the first four weeks of hospitalisation were analyzed; a total of 4,394 patients in the analytic sample were excluded from this analysis. We assessed the association between history of vaccination and in-hospital death using mixed effects logistic regression, including as covariates age, sex and presence of comorbidities, and random effects for different countries; age was included as a binary variable (age below versus above 60 years). Qualitatively similar results, not presented here, were obtained when using Cox proportional hazards models stratified by country.

R and Python were used for data processing and descriptive analyses, and Stata 17 was used to fit mixed effects logistic regression models.

## Data Availability

• The data that underpin this analysis are highly detailed clinical data on individuals hospitalised with COVID-19. Due to the sensitive nature of these data and the associated privacy concerns, they are available via a governed data access mechanism following review of a data access committee. Data can be requested via the IDDO COVID-19 Data Sharing Platform (http://www.iddo.org/covid-19). The Data Access Application, Terms of Access and details of the Data Access Committee are available on the website. Briefly, the requirements for access are a request from a qualified researcher working with a legal entity who have a health and/or research remit; a scientifically valid reason for data access which adheres to appropriate ethical principles. The full terms are at https://www.iddo.org/document/covid-19-data-access-guidelines. A small subset of sites who contributed data to this analysis have not agreed to pooled data sharing as above. In the case of requiring access to these data, please contact the corresponding author in the first instance who will look to facilitate access. Code used in the descriptive analysis is available. • Any additional information required to reanalyze the data reported in this paper is available from the lead contact upon request.

## Supplemental information

Document S1. Figures S1–S4 and Tables S1–S10

Data S1. Supplemental results, related to STAR Methods and Figures 1–5

Document S2. Article plus supplemental information
