# Supplementary material for: A multi-country analysis of COVID-19 hospitalizations by vaccination status
Source: Med. 2023 Nov 10;4(11):797–812.e2. doi: 10.1016/j.medj.2023.08.005 (PMC10935543; doi:10.1016/j.medj.2023.08.005)
Supplement: Document S1. Figures S1–S4 and Tables S1–S10 [file mmc1.pdf]

Med, Volume *4*

Supplemental information

A multi-country analysis of COVID-19 hospitalizations by vaccination status

Bronner P. Gonçalves, Waasila Jassat, Joaquín Baruch, Madiha Hashmi, Amanda Rojek, Abhishek Dasgupta, Ignacio Martin-Loeches, Luis Felipe Reyes, Chiara Piubelli, Barbara Wanjiru Citarella, Christiana Kartsonaki, Benjamin Lefèvre, José W. López Revilla, Miles Lunn, Ewen M. Harrison, Moritz U.G. Kraemer, Sally Shrapnel, Peter Horby, Zeno Bisoffi, Piero L. Olliaro, Laura Merson, and ISARIC Clinical Characterisation Group

Supplementary Appendix

Title

A multi-country analysis of COVID-19 hospitalisations by vaccination status

Summary

Supplementary Figures (Figures S1-S4)
Supplementary Tables (Tables S1-S10)

Supplementary figures

Figure S1. Vaccination coverage and relative frequencies of SARS-CoV-2 variants in countries contributing data to this study, related to Figure 1. Data used to generate this figure are publicly available, and described in the *Methods* section. Only countries with 100 or more patients in the analytic sample were included. In each panel, the dashed black line corresponds to vaccine coverage, and coloured lines correspond to relative frequencies of the four variants in this analysis. The coloured areas represent periods when SARS-CoV-2 variants were dominant, here defined as causing 90% or more of infections in the genomic database, GISAID; only epidemiological weeks with at least 10 samples informing country-specific variant composition were analysed, and after the start of each variant period, drops in frequencies to the interval between 80 and 90% were not considered in defining the end of the period.

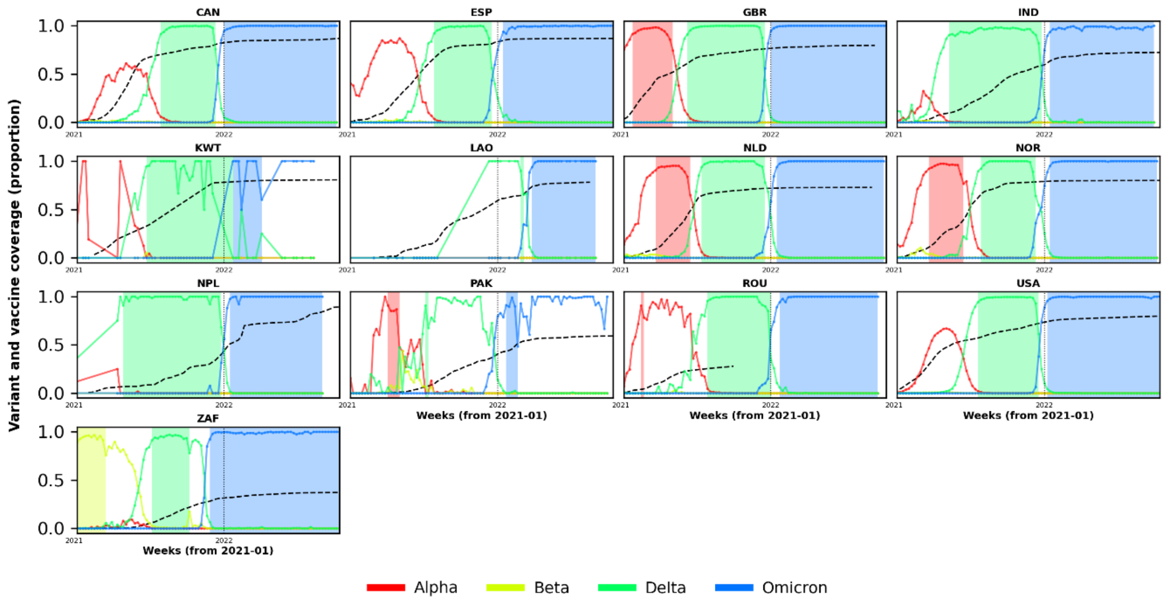

Figure S2. Frequency of vaccination history by 4-week period and country, related to Figure 2. Proportions of hospitalised study participants with previous vaccination are presented in blue. Only countries with at least 100 observations were included; four-week periods with at least 5 participants are presented. Four-week periods with less than 20 participants have a star.

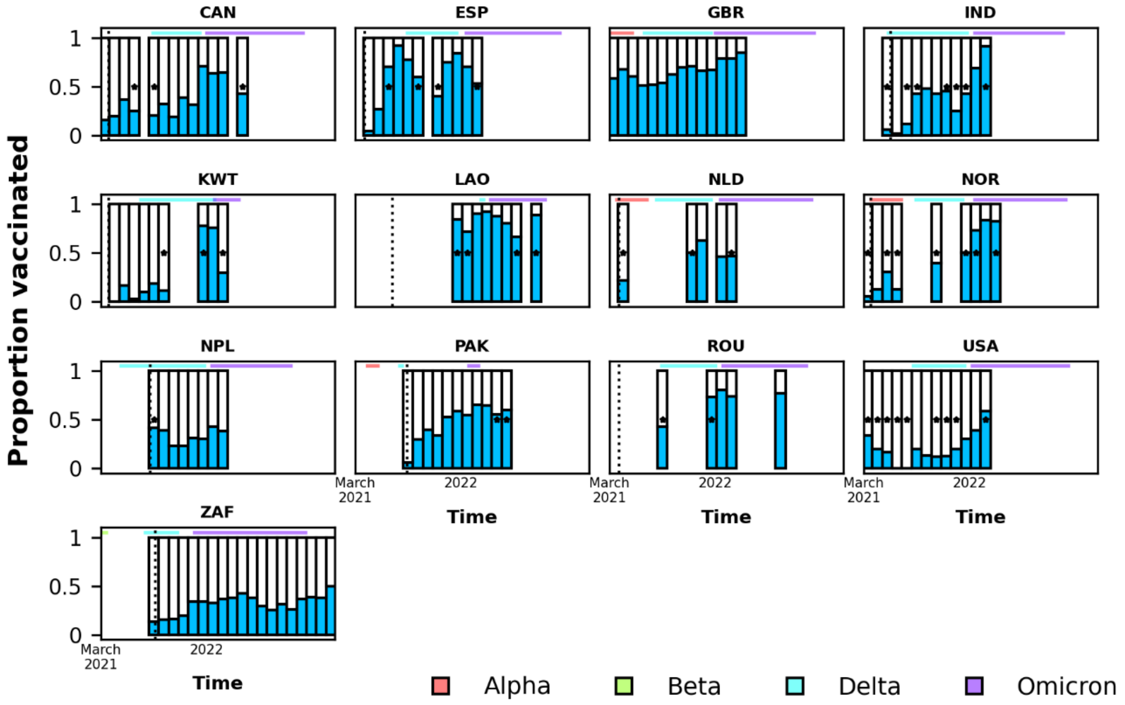

Figure S3. Differences in frequencies of comorbidities between vaccinated and unvaccinated groups, related to Figure 4. This figure is similar to **Figure 4** except for the quantity represented by the colours in each square. Here, each difference in the frequencies of comorbidities between unvaccinated and vaccinated patients is presented relative to frequencies in the unvaccinated. By definition, the highest value of this quantity is 1; values below -1, i.e. frequencies in vaccinated patients at least twice as high as those in unvaccinated patients, were set to -1 to facilitate visualisation of less extreme relative differences. For the same reason, the range of the colour scale is different from that in **Figure 4**. Note that in this figure, country-specific relative differences are not presented when the corresponding comorbidities were not reported in the unvaccinated group - *i.e.* when the denominator in the calculation would be zero.

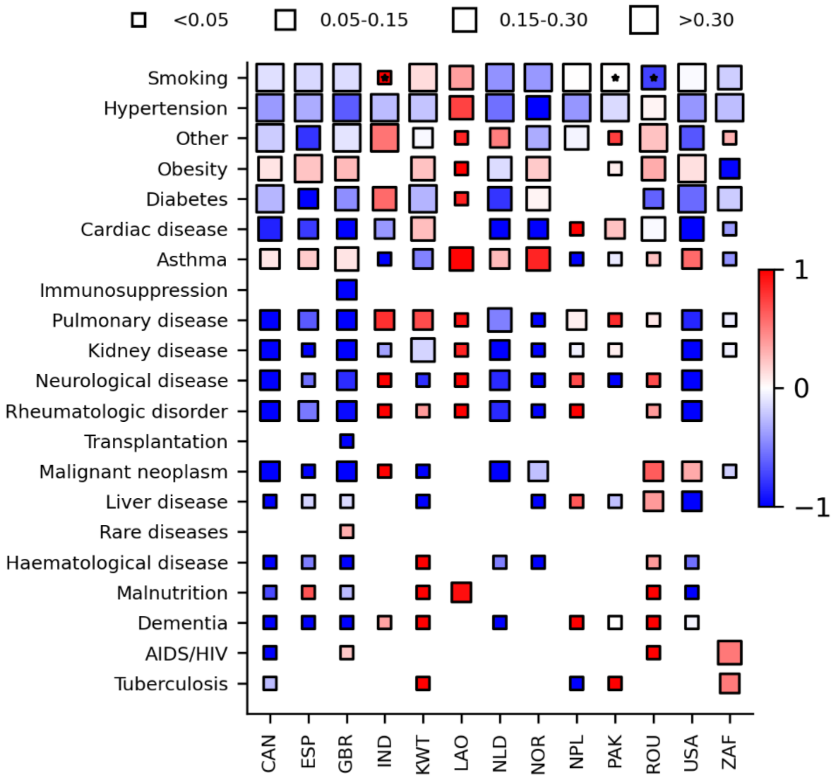

Figure S4. Fatality risks in vaccinated (y-axis) and unvaccinated (x-axis) patients by country, represented by different colours, and age; related to Figure 5. This figure is similar to **Figure 5** in the main text, except for the range of the two axes. This version of the figure allows better visualisation of fatality risks in settings with relatively lower values. Data from countries with fatality risk above 0.30 in either group, vaccinated or unvaccinated, are not shown; note for some data points entire confidence intervals are only presented in **Figure 5**.

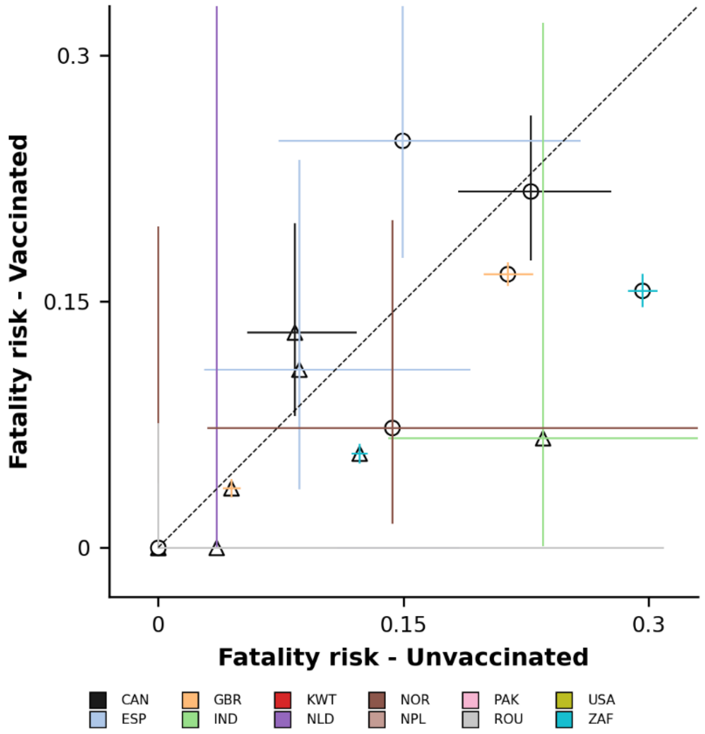

Supplementary tables

Table S1. Percentages of participants included in the analytic sample that required intensive care; related to the STAR Methods . Countries highlighted in blue had more than 80% of study patients admitted to intensive care; 1,791 records had missing information on ICU admission. Only countries with at least 10 patients are presented.

Country	Percentage
Argentina	100.0
India	100.0
United Arab Emirates	100.0
Qatar	100.0
Portugal	100.0
Pakistan	100.0
Austria	100.0
Nepal	100.0
Germany	100.0
China	100.0
Estonia	100.0
Kuwait	98.6
United States	97.0
New Zealand	95.9
Saudi Arabia	93.8
Colombia	90.8
Italy	89.8
Malaysia	69.4
Spain	66.7
Brazil	64.6
Canada	49.2
Indonesia	27.6
Norway	23.1
Uganda	18.2
Israel	16.7
Turkey	16.3
Netherlands	15.4
United Kingdom	14.4
South Africa	8.4
Bolivia	7.7
Philippines	6.3
Lao PDR	4.8
Romania	0.5
Malawi	0.0
Ireland	0.0

Table S2. Frequency of previous vaccination in the analytic sample by country, related to Figure 2. Countries with at least 10 participants are included.

Country	Total N	% vaccinated
Argentina	43	27.9
Austria	34	2.9
Bolivia	13	30.8
Brazil	48	79.2
Canada	1297	44.4
China	40	57.5
Colombia	65	32.3
Estonia	26	3.8
Germany	14	7.1
India	233	34.3
Indonesia	29	69.0
Ireland	27	14.8
Israel	18	50.0
Italy	49	8.2
Kuwait	208	21.6
Lao PDR	393	87.5
Malawi	20	35.0
Malaysia	98	84.7
Nepal	680	32.2
Netherlands	136	50.0
New Zealand	76	44.7
Norway	156	44.9
Pakistan	1086	44.5
Philippines	66	78.8
Portugal	40	10.0
Qatar	19	5.3
Romania	198	76.8
Saudi Arabia	16	37.5
South Africa	47768	27.6
Spain	312	59.9
Turkey	49	73.5
Uganda	11	45.5
United Arab Emirates	15	13.3
United Kingdom	29637	64.2
United States	232	24.1

Table S3. Age distribution by vaccination status and country, related to Figures 2 and 3, and to the STAR Methods. In **Table S3A**, median ages of vaccinated and unvaccinated patients by country are presented. Only countries contributing data on at least 100 participants are included in this table. Table S3B includes the percentages of patients aged 60 years or older, by vaccination status, country and calendar year. Percentages based on 20 or fewer observations are highlighted in orange. Only countries with at least 100 patients included in the analytic sample are presented.

Table S3A

Country	Median (IQR) age in years	
	Vaccinated	*Unvaccinated*
Canada	70 (59 - 81)	60 (48 - 72)
India	64 (56 - 71)	57 (41 - 69)
Kuwait	57 (47 - 67)	48 (39 - 61)
Lao PDR	34 (27 - 42)	52 (36 - 70)
Nepal	64 (50 - 74)	58 (42 - 74)
Netherlands	75 (69 - 80)	57 (48 - 73)
Norway	73 (57 - 80)	51 (44 - 61)
Pakistan	64 (50 - 73)	62 (50 - 73)
Romania	52 (40 - 64)	54 (45 - 60)
South Africa	57 (43 - 71)	49 (34 - 65)
Spain	69 (60 - 77)	62 (50 - 69)
United Kingdom	72 (57 - 82)	49 (37 - 63)
United States	66 (58 - 73)	57 (46 - 66)

Table S3B

Country	2021		2022	
	Vaccinated	*Unvaccinated*	*Vaccinated*	*Unvaccinated*
Canada	76.1	50.2	69.5	56.9
India	53.3	39.4	80.0	87.5
Kuwait	34.8	26.8	50.0	64.3
Lao PDR	5.3	66.7	6.8	37.0
Nepal	62.6	44.9	66.3	59.6
Netherlands	90.2	56.8	85.2	35.5
Norway	59.1	24.3	77.1	75.0
Pakistan	58.2	56.2	64.3	70.8
Romania	30.8	50.0	32.4	21.1
South Africa	41.2	33.7	47.5	34.3
Spain	75.4	52.0	75.4	60.0
United Kingdom	70.3	29.2	75.5	50.4
United States	77.4	41.5	68.0	55.2

Table S4. Frequencies of symptoms in vaccinated and unvaccinated patients, related to Figure 3. **Table S4A** presents frequencies of different symptoms by vaccination status in the combined dataset; data from South Africa are not included. Of participants with data on at least one symptom, vaccinated patients reported a median of 3 different symptoms (IQR, 2 – 5), whilst unvaccinated patients had a median of 4 symptoms (IQR, 2 – 5). Considering only the ICU country group, the median numbers of symptoms in vaccinated and unvaccinated patients were similar, 3 (IQR 2 – 4). URT, upper respiratory tract. In **Table S4B**, percentages of participants with at least one of the five most common symptoms are presented by vaccination status for the different SARS-CoV-2 variant periods. Only patients with data on all five symptoms (see *Results* section) are included.

Table S4A

Symptoms	Vaccinated			Unvaccinated		
	%	Total (non-missing)	Missing data	%	Total (non-missing)	Missing data
Abdominal pain	9.6	14146	7519	8.9	10669	3061
Confusion	20.2	14434	7231	11.2	10747	2983
Bleeding	1.8	14036	7629	1.2	10570	3160
Chest pain	17.0	14494	7171	22.9	10958	2772
Conjunctivitis	0.2	13542	8123	0.2	10229	3501
Cough	64.7	15345	6320	74.2	11576	2154
Diarrhoea	15.9	14433	7232	19.9	10905	2825
Ear pain	0.3	11465	10200	0.5	8262	5468
Fatigue/Malaise	42.6	14084	7581	45.8	10692	3038
Headache	14.0	13525	8140	17.7	10340	3390
Fever	51.2	14942	6723	67.1	11330	2400
Altered smell	6.7	12904	8761	11.6	9835	3895
Altered taste	8.1	12744	8921	13.2	9714	4016
Lymphadenopathy	0.4	13582	8083	0.4	10275	3455
Muscle/joint pain	16.7	13344	8321	23.7	10283	3447
Runny nose	5.2	13126	8539	3.7	9908	3822
Seizures	1.0	14096	7569	0.8	10612	3118
Severe dehydration	12.1	8356	13309	10.4	4951	8779
Shortness of breath	63.8	15480	6185	76.2	11659	2071
Skin rash	1.3	13801	7864	1.1	10448	3282
Sore throat	9.2	13154	8511	10.0	9964	3766
Vomiting nausea	20.1	14492	7173	24.0	10969	2761
Wheezing	7.6	13703	7962	5.5	10285	3445
URT symptoms	11.5	13428	8237	11.8	10128	3602

Table S4B

Variant	Vaccinated		Unvaccinated	
	Total	*% with symptoms*	*Total*	*% with symptoms*
Alpha	822	76.6	680	91.3
Delta	7500	93.8	6100	96.8
Omicron	3242	87.1	1312	90.7

Table S5. Additional information on comorbidities and clinical outcomes, related to Figure 4 and Figure 5. **Table S5A** includes the percentages of patients with 3 or more comorbidities by vaccination status and variant-defined epidemiological period. **Table S5B** shows percentages of patients who died in the first 28 days after hospital admission or disease onset, whichever happened later. Criteria for inclusion in these calculations are described in the *Methods* section. **Table S5C** shows presents the frequency of the composite outcome by country group, age and vaccination status.

Table S5A

**Variant**	***Vaccinated***		***Unvaccinated***	
	% with 3 or more comorbidities	Total	% with 3 or more comorbidities	Total
Alpha	70.1	1082	33.2	749
Delta	54.4	11777	16.8	14186
Omicron	27.7	12216	11.0	12785

Table S5B

**Country**	**Total**	**% 28-day fatality**
Romania	182	0.0
Norway	114	5.3
United Kingdom	27984	11.7
South Africa	46695	15.9
Canada	1143	17.4
Spain	304	17.4
Netherlands	108	26.9
India	175	29.7
United States	228	34.6
Kuwait	196	45.9
Pakistan	702	49.4
Nepal	273	65.2

Table S5C

Countries with less than 80% ICU admission					
		**Unvaccinated**		**Vaccinated**	
	Age (years)	*Total*	*% composite outcome*	*Total*	*% composite outcome*
	18-60	29747	14.1	12814	8.1
	>60	14896	32.0	18412	20.8
ICU country group					
		**Unvaccinated**		**Vaccinated**	
	Age (years)	*Total*	*% composite outcome*	*Total*	*% composite outcome*
	18-60	941	88.1	367	90.7
	>60	887	91.4	591	90.9

Table S6. Mixed effects logistic models on death in the first 28 days after admission or disease onset, related to Figure 5. We present results for three models. For model III, that includes number of comorbidities as a covariate, only patients with data on at least 10 comorbidity variables were included (N = 54,738); the number of individuals analysed in models I and II were 78,769 and 78,733, respectively. In addition to adjusting for coexisting medical conditions by including number of comorbidities as a covariate, we also fit a model that included instead a binary variable defined based on whether patients had three or more comorbidities; the estimated odds ratio for the association between vaccination and death outcome was similar to the odds ratios in the table..

**Model**		**I**	**II**	**III**
**Variables**		**Odds ratio (95% CI)**	**Odds ratio (95% CI)**	**Odds ratio (95% CI)**
	Previous vaccination	0.77 (0.74 - 0.80)	0.59 (0.56 - 0.62)	0.53 (0.50 - 0.56)
	Sex (Female)	-	0.87 (0.84 - 0.91)	0.85 (0.81 - 0.90)
	Age			
	Aged between 18 and 60 years	-	Reference	Reference
	Older than 60 years	-	3.41 (3.27 - 3.57)	2.89 (2.73 – 3.05)
	Number of comorbidities	-	-	1.25 (1.23 – 1.27)

Table S7. Sensitivity analysis I, that includes data from all records from March 2021 regardless of country-level vaccination coverage, related to Table 2 and Figure 5. **Table S7A** includes information on multiple comorbidities; and **Table S7B**, on fatality risk.

Table S7A

Country	*Vaccinated*		*Unvaccinated*	
	% with 3 or more comorbidities	Total	% with 3 or more comorbidities	Total
Canada	63.1	563	40.0	732
India	13.6	88	19.9	362
Kuwait	26.1	46	27.6	181
Lao PDR	0.6	344	14.3	49
Malawi	5.3	38	7.1	170
Nepal	5.5	219	4.5	463
Netherlands	62.5	72	37.2	113
Norway	58.9	73	27.0	115
Pakistan	1.0	488	1.3	628
Peru	-	< 10	6.4	204
Philippines	32.1	53	20.5	122
Romania	12.5	152	15.2	46
South Africa	7.7	11685	7.9	39298
Spain	50.8	187	30.0	150
United Kingdom	65.3	15450	37.1	6642
United States	78.6	56	50.9	175

Table S7B

Country	Total	% 28-day fatality
Romania	182	0.0
Philippines	170	2.4
Peru	175	3.4
Norway	125	5.6
United Kingdom	27984	11.7
Malawi	171	11.7
Spain	327	16.8
Canada	1178	17.2
South Africa	80225	20.6
Netherlands	157	24.8
Colombia	101	27.7
India	388	30.2
United States	228	34.6
Kuwait	214	44.9
Pakistan	730	48.8
Nepal	275	64.7

Table S8. Sensitivity analysis II, that uses a vaccination coverage of 20%, rather than 10%, relates to Table 2 and Figure 5. Table S8A includes information on multiple comorbidities; and **Table S8B**, on fatality risk.

Table S8A

Country	*Vaccinated*		*Unvaccinated*	
	% with 3 or more comorbidities	Total	% with 3 or more comorbidities	Total
Canada	65.3	490	41.9	382
India	7.8	77	9.6	83
Kuwait	28.2	39	42.5	73
Lao PDR	0.6	344	14.3	49
Nepal	5.2	172	4.6	388
Netherlands	64.1	64	26.9	52
Norway	58.2	67	27.8	54
Pakistan	1.1	440	1.4	443
Romania	12.5	152	15.2	46
South Africa	7.5	7817	7.4	13020
Spain	50.8	187	24.0	96
United Kingdom	65.3	15450	37.1	6642
United States	78.6	56	50.9	175

Table S8B

Country	Total	% 28-day fatality
Romania	182	0.0
Norway	101	5.0
United Kingdom	27984	11.7
South Africa	32429	12.4
Spain	275	17.8
Canada	812	19.7
India	102	24.5
United States	228	34.6
Kuwait	104	45.2
Pakistan	542	48.3
Nepal	230	64.8

Table S9. Frequency of symptoms in the subset of dataset that includes only patients admitted to hospital before March 2021, related to Figure 3. URT, upper respiratory tract.

Symptoms	%	Total (non-missing)	Missing data
Abdominal pain	8.2	189557	52057
Confusion	19.3	192340	49274
Bleeding	1.6	186198	55416
Chest pain	12.5	193203	48411
Conjunctivitis	0.3	181292	60322
Cough	56.6	207817	33797
Diarrhoea	15.6	195943	45671
Ear pain	0.3	151125	90489
Fatigue/Malaise	38.4	189812	51802
Headache	10.7	183150	58464
Fever	55.9	207805	33809
Altered smell	6.7	149454	92160
Altered taste	7.9	146449	95165
Lymphadenopathy	0.5	170118	71496
Muscle/joint pain	17.6	182933	58681
Runny nose	3.0	177037	64577
Seizures	1.0	185116	56498
Severe dehydration	12.3	83798	157816
Shortness of breath	59.1	208651	32963
Skin rash	2.2	182858	58756
Sore throat	7.0	177129	64485
Vomiting nausea	15.6	195379	46235
Wheezing	5.9	181987	59627
URT symptoms	9.0	182199	59415

Table S10. Country-specific percentages of patients admitted to hospital before March 2021 who had 3 or more comorbidities, related Table 2. Only patients with data on at least ten comorbidity-related variables are included in this table.

Country	% with 3 or more comorbidities	Total
Argentina	35.0	100
Australia	18.9	424
Belgium	47.0	779
Brazil	34.0	3164
Cameroon	0.6	179
Canada	59.9	3500
Chile	36.0	111
Colombia	27.7	444
France	41.6	4612
Germany	39.7	116
Ghana	0.8	2453
Gibraltar	5.3	394
Guinea	1.4	1086
India	14.9	3262
Indonesia	30.0	701
Ireland	45.7	1305
Israel	14.4	1021
Italy	34.1	3133
Kuwait	29.0	563
Malaysia	6.8	5737
Nepal	2.8	959
Netherlands	35.8	1871
Norway	8.0	3193
Pakistan	7.6	4671
Peru	4.9	1340
Philippines	15.1	106
Portugal	46.8	1037
Qatar	11.8	306
Romania	14.1	722
Russian Federation	31.3	1658
Senegal	3.8	133
South Africa	9.7	133204
Spain	14.4	11652
Uganda	3.3	212
Ukraine	63.1	103
United Arab Emirates	21.4	145
United Kingdom	56.0	157894
United States	46.7	4118
