# Supplementary material for: A multi-country analysis of COVID-19 hospitalizations by vaccination status
Source: Med. 2023 Nov 10;4(11):797–812.e2. doi: 10.1016/j.medj.2023.08.005 (PMC10935543; doi:10.1016/j.medj.2023.08.005)
Supplement: Data S1. Supplemental results, related to STAR Methods and Figures 1–5 [file mmc2.zip › Data S1.docx]

**Data S1. This file contains Supplementary Results, related to STAR Methods, Figures 1-5.**

*Comparison of patients by availability of information on vaccination status*

For patients admitted to hospital on or after March 2021 (see **Figure 1**), data on previous vaccination were missing for 26,865 and reported as unknown for 178,539. Here we compare participants with known vaccination status and the combined group with either missing information or unknown vaccination status.

Patients in both groups, those with known and unknown/missing vaccination history, were more often admitted in 2021 (80.3% and 79.5%, respectively), slightly more likely to be female (52.1% and 50.4%), and more often younger than 60 years (56.6% and 54.8%). Patients with known vaccination status were slightly more likely to have 3 or more comorbidities compared to patients with missing information on or unknown vaccination history (22.8% versus 19.7%). Patients with known vaccination status had fatality risk (18.6%) similar to that of patients with missing data or unknown vaccination status (18.8%).

*Composite outcome of invasive mechanical ventilation or death*

Data on invasive mechanical ventilation (IMV) were available for 82,033 patients in the analytic sample; of these, 6,450 required IMV. Information on the date of initiation of IMV was only available for 2,428/6,450 patients; hence time since admission was not used in defining the composite outcome. 3,378 participants who did not require IMV were excluded because they had unknown clinical outcome, including related to ongoing care and transfer. Amongst patients with IMV data, 16,350/78,655 (20.8%) had the composite outcome of either IMV or death. Considering only the ICU country group, a high percentage (90.1%; 2,510/2,786) of patients had the composite outcome. For patients recruited in countries with frequency of ICU admission below 80%, a lower percentage had composite outcome (18.2%; 13,840/75,869). **Table S5** presents frequencies of the composite outcome in these two groups of countries, by vaccination status and age category.

*Sensitivity analyses using a different threshold for country-specific vaccine coverage*

In our primary analysis, we analysed data from hospitalised COVID-19 patients admitted after vaccination coverage reached 10%. The rationale for this was to avoid comparing vaccinated and unvaccinated patients admitted in non-overlapping time periods. Below we present results of two different sensitivity analyses: one that did not apply this coverage-based inclusion criterion, and another that uses the coverage value of 20% as threshold.

Sensitivity analysis I

When all records of patients hospitalised on or after March 2021 are included in the analysis, the median age in the vaccinated group is 65 (50 – 78), and in the unvaccinated group, 53 (38 – 66). The frequency of three or more comorbidities in the vaccinated group was 39.5%, compared to 12.8% in the unvaccinated group; **Table S7** presents this information by country, and as can be seen, results in that table are not dissimilar to those in the **Table 2**. **Table S7** also presents the fatality risks by country and vaccination status, which are in general similar to those in **Table S5**; note that in South Africa, the risk of death was higher in this analysis that did not apply a coverage threshold.

Sensitivity analysis II

Using a higher vaccine coverage threshold (20%) led to inclusion of 67,393 patients in the analysis. **Table S8** presents country-specific data on comorbidities and fatality risk. As above, country-specific differences between vaccinated and unvaccinated patients are consistent with those discussed in the main manuscript.

*Pre-vaccination data*

In this section, for completeness, we briefly report a general description of the data from patients admitted before March 2021 (N=441,842 participants). The median of the ages of patients recruited during the pre-vaccination data period was 61 years, and the interquartile range, 25 – 92 years; 51.9% were male. **Table S9** shows frequencies of symptoms for participants in this subset of our dataset. Of those patients who had data on at least ten comorbidities (N=357,259), 32.7% had three or more comorbidities (see also **Table S10** for corresponding country-specific percentages).
